# Structural Basis Underlying the Binding Preference of Human Galectins-1, -3 and -7 for Galβ1-3/4GlcNAc

**DOI:** 10.1371/journal.pone.0125946

**Published:** 2015-05-06

**Authors:** Tung-Ju Hsieh, Hsien-Ya Lin, Zhijay Tu, Bo-Shun Huang, Shang-Chuen Wu, Chun-Hung Lin

**Affiliations:** 1 Institute of Biological Chemistry, Academia Sinica, Taipei, Taiwan; 2 The Genomics Research Center, Academia Sinica, Taipei, Taiwan; 3 Department of Chemistry, National Taiwan University, Taipei, Taiwan; 4 Institute of Biochemical Sciences, National Taiwan University, Taipei, Taiwan; INRS, CANADA

## Abstract

Galectins represent β-galactoside-binding proteins and are known to bind Galβ1-3/4GlcNAc disaccharides (abbreviated as LN1 and LN2, respectively). Despite high sequence and structural homology shared by the carbohydrate recognition domain (CRD) of all galectin members, how each galectin displays different sugar-binding specificity still remains ambiguous. Herein we provided the first structural evidence of human galectins-1, 3-CRD and 7 in complex with LN1. Galectins-1 and 3 were shown to have higher affinity for LN2 than for LN1, while galectin-7 displayed the reversed specificity. In comparison with the previous LN2-complexed structures, the results indicated that the average glycosidic torsion angle of galectin-bound LN1 (ψ^LN1^ ≈ 135°) was significantly differed from that of galectin-bound LN2 (ψ^LN2^ ≈ -108°), i.e. the GlcNAc moiety adopted a different orientation to maintain essential interactions. Furthermore, we also identified an Arg-Asp/Glu-Glu-Arg salt-bridge network and the corresponding loop (to position the second Asp/Glu residue) critical for the LN1/2-binding preference.

## Introduction

Galectins, β-galactoside-binding proteins, are characteristic of having one or two conserved carbohydrate recognition domains (CRDs) [1, 2]. Members of this family have been shown to participate in diverse biological functions, such as cell adhesion, cell growth regulation, and apoptosis via their interactions with β-galactoside-containing structures on cell surface, e.g., N-, O-linked glycoproteins, proteoglycans or glycolipids [3, 4]. More importantly, human galectins act as regulatory factors in many types of cancers by either inhibiting or promoting tumor growth [5]. Therefore, to identify selective ligands for human galectins provides not only a useful tool for dissecting how each galectin member interacts with specific glycan structures in correlation with cancer progression, but also a possible solution for the development of clinical therapeutics.

All galectins are able to recognize Galβ1-3/4GlcNAc disaccharides, namely type 1 and 2 LacNAc (abbreviated as LN1 and LN2, respectively) that appear in a myriad of glycoconjugates. For instance, LN1 and LN2 are shown as the repeating structures in the non-reducing termini of lacto-series glycans, including blood group antigens. LN2 are constitutively expressed in all mammalian cell types, while LN1 are more tissue-specifically distributed mostly restricted to the epithelia of gastrointestinal and reproductive tract in humans [6]. Interestingly, the presence of LN1 but not the LN2 was recently found to be in association with the pluripotency of induced pluripotent stem cells (iPSCs) [7, 8]. Furthermore, studies on the chemical structures of the milk oligosaccharides produced by various mammalian species revealed that LN1-containing oligosaccharides predominate over LN2-containing in human milk [9]. It has been hypothesized to be a selective advantage for human that the acquisition of predominantly LN1-containing oligosaccharides may promote the growth of specific anti-pathogenic bifidobacteria in the infant colon and thus aid their survival [9].

Galectins share major structural homology in their CRDs, but different galectin members were shown to display deviated binding preference for LN1- and LN2-containing glycans [10–13]. Albeit several crystal structures of galectin/LN2 complexes are available, there is no report regarding to the structure of galectin/LN1 complex. Therefore, the molecular basis still remains enigmatic to underlie the distinct binding preferences of galectins for LN1/2.

To bridge this gap, we herein report the crystal structures including full-length human galectin-1 (hGal1), galectin-7 (hGal7) and the C-terminal CRD domain of galectin-3 (hGal3-CRD) in complex with LN1. In comparison with the analogous LN2-complexed structures (deposited in Protein Data Bank with ID codes of 1W6P [14], 1KJL [15], 5GAL [16] for hGal1, 3-CRD and 7, respectively), the results pinpoint that in the bound LN1 and LN2, N-acetylglucosamine moiety adopts a different orientation while the galactose keeps the same posture. Additionally, we identified a unique Arg-Asp/Glu-Glu-Arg salt-bridges network (Arg48^hGal1^–Asp54^hGal1^–Glu71^hGal1^Arg73^hGal1^, Arg162^hGal3^–Glu165^hGal3^–Glu184^hGal3^–Arg186^hGal3^ and Arg53^hGal7^–Glu58^hGal7^–Glu72^hGal7^–Arg74^hGal7^) to be essential for the LN1- or LN2-binding preferences. The loop harboring Asp54^hGal1^, Glu165^hGal3^ or Glu58^hGal7^ was found to affect how the salt-bridge is arranged and associated with possible water-mediated interactions.

## Materials and Methods

### Protein preparation

Using a standard PCR-based cloning strategy, the coding region of the full-length hGal1 (residues 1–135), 3 (residues 1–250), 7 (residues 1–136) and CRD domain of hGal3 (residues 113–250) were generated and inserted into modified pET-15b (hGal1, hGal3 and hGal3-CRD) or pET-28a (hGal7) vector (Novagen) with in-frame N- and C-terminal 6xHis-tag, respectively. QuikChange mutagenesis method (Agilent Technologies) was applied to replace the corresponding codons of residues Glu165 and Arg186 in full-length hGal3 with Ala codon to allow the expression of mutant proteins hGal3-E165A and hGal3-R186A. All the wild type and mutated proteins were produced in *Escherichia coli* BL21 (DE3), with 0.5 mM IPTG induction for 16 h at 20 °C. Recombinant proteins were purified by Ni^2+^-affinity and size-exclusion chromatography to homogeneity. Purified proteins were then stored in gel-filtration buffer (25 mM Tris-HCl pH8.0, 300 mM NaCl and 5 mM β-mercaptoethanol) and concentrated to ~ 4, 20 and 9 mg/ml as determined by the method of Bradford for both Biolayer interferometry experiments and crystallization trials, respectively.

### Biolayer interferometry

LN1 and LN2 binding affinity of hGal1, hGal3 and hGal7 were quantitatively measured in 96-well microplates at 27 °C by Octet Red system (FortéBio). Specifically, biotinylated galectins were prepared according to the standard protocol (provided by FortéBio), and adjusted to final concentration of 1 μM in assay buffer condition (50 mM Tris-HCl pH 7.5, 300 mM NaCl and 5 mM β-mercaptoethanol). Biotinylated galectins were then immobilized on Super Streptavidin Biosensors (FortéBio, Inc.), while free streptavidin sites on biosensor were then blocked by incubation with biocytin (10 mg/ml) to avoid non-specific interactions. The assay was carried out by placing galectin-coated biosensors into the wells with a concentration series of 3-fold diluted LN1/LN2 solutions (200 μl per well, from a top concentration of 3 mM for hGal1 and hGal7, and 1 mM for hGal3) and measuring changes in layer thickness (in nanometers) of biosensors with time. Measurements were followed by 120 sec baseline step, 60 sec association step and 200 sec dissociation step. Baseline and dissociation steps were carried out in assay buffer only. All the data were processed and calculated using Fortebio software and the steady-state *K*_*d*_ values were derived from equilibrium responses (S1 Fig) and summarized in Table 1. Two additional parallel Super Streptavidin biosensors were coated with biotinylated galectin and biocytin, separately, and only incubated with assay buffer as double reference controls.

**Table 1:** Dissociation constants (*K*_*d*_ in μM) of human galectins-1, 3, 7 and mutants for Galβ1-3/4GlcNAc disaccharides^1^,^2^.

	*K*_*d*_ (μM)	
Protein	Galβ1-3GlcNAc (LN1)	Galβ1-4GlcNAc (LN2)
hGal1	340^1^ / 6.74 ± 3.14^2^	150^1^ / 3.59 ± 2.35^2^
hGal3	93^1^ / 1.44 ± 0.28^2^	33^1^ / 0.33 ± 0.064^2^
hGal7	270^1^ / 6.63 ± 1.27^2^	410^1^ / 19.69 ±3.45^2^
hGal3-E165A	230^1^	280^1^
hGal3-R186A	ND^3^	ND^3^

^1^The values are determined by Biolayer Interferometry at 300 K.

^2^The values are determined by fluorescence anisotropy at 277 K.

^3^The binding is too weak to be determined by Biolayer Interferometry.

### Fluorescence polarization (FP)-based competition binding assay

The measurement of FP-based assay is based on the rotation speed of a fluorophore-containing compound bound to the protein counterpart (e.g. galectins in this study). The fluorophore rotates at a slower rate than when it is unbound, and the resulting fluorescence polarization is higher. In this study we carried out all measurements according to reported procedures [17, 18]. To the final sample volume (70 μl) in each assay was added a synthetic FITC-conjugated type 2 LacNAc (LN2-FITC; as the fluorescent probe or reference compound) to a final concentration of 0.1 μM. All the measurements were conducted in Tris buffer (12.5 mM, pH 7.4) with 200 mM NaCl and 5 mM β-mercaptoethanol at 4 °C. Data plotting, nonlinear regression analysis, and curve construction was done by Prism 5.0 software (GraphPad, San Diego, CA).

For direct binding assay of LN2-FITC, the data (anisotropy, A vs. hGal 1, 3 and 7 concentration, respectively) were fitted to the formula $\text{A}={\text{A}}_{0}+{\text{A}}_{\text{max}}\times \left[\text{hGal}\right]\;∕\;\left(K_{d}+\left[\text{hGal}\right]\right)$ to estimate *K*_*d*_^*Probe*^ value (S2 Fig), where A_0_ is the anisotropy value measured in the absence of hGal and A_max_ is the maximum value approached with increasing [hGal].

For FP-based competition assay, commercial LN1 (or LN2) molecules (Dextra, UK) at indicated concentration 300, 6 and 300 μM were used as competitors to compete binding interaction between 0.1 μM fluorescent probe (LN2-FITC) and selected concentration of hGal1 (120 μM), 3 (3 μM) and 7 (120 μM), respectively. The measured anisotropy value with competitor (A_competitor_) is used to calculate the amount of galectin-bound probe [PG] according to equation:

$$\left[\text{PG}\right]=\left[\left({\text{A}}_{\text{competitor}}-{\text{A}}_{0}\right)\;∕\;\left({\text{A}}_{\text{max}}-{\text{A}}_{0}\right)\right]\times {\left[\text{P}\right]}_{\text{total}}.-$$

And therefore, *K*_*d*_^*Competitor*^ value was further deduced by the following equations and summarized in Table 1:

$$\begin{array}{l} \left[\text{P}\right]={\left[\text{P}\right]}_{\text{total}}-\left[\text{PG}\right]-\\ \left[\text{G}\right]=K_{d}^{Probe}\times \left[\text{PG}\right]\;∕\;\left[\text{P}\right]-\\ \left[\text{CG}\right]={\left[\text{G}\right]}_{\text{total}}-\left[\text{PG}\right]-\left[\text{G}\right]-\\ \left[\text{C}\right]={\left[\text{C}\right]}_{\text{total}}-\left[\text{CG}\right]-\\ K_{d}^{Competitor}=\left[\text{C}\right]\times \left[\text{G}\right]∕\left[\text{CG}\right].- \end{array}$$

[C], [P] and [G] are the concentrations of free competitor, probe, and galectins, respectively, and [CG] and [PG] are the concentrations of competitor-galectin complex and the probe-galectin complex, respectively.

### Crystallization and data collection

Crystals of recombinant hGal1, 3-CRD were grown at room temperature (298 K) using the hanging-drop vapor diffusion method from 2 μl protein solution and 2 μl reservoir solutions consisting of 0.1 M Tris pH 8.0, 0.2 M Li_2_SO_4_, 30% (w/v) PEG 3350 and of 0.1 M Tris pH 8.5, 0.2 M MgCl_2_, 30% (w/v) PEG 4000, for hGal1 and hGal3-CRD respectively. The hGal1-LN1 and hGal3-CRD-LN1 complexes were obtained by soaking their native protein crystals with 20 mM type I N-acetyllactosamine (LN1) for more than a week. hGal7 protein solution at 9 mg/ml was incubated overnight with 2 mM LN1 molecule at 4 °C. Following overnight incubation, the protein-ligand mixtures were centrifuged at 13,000 rpm for 5 min to remove the precipitated protein. Cocrystals for the hGal7-LN1 complexes were carried out by the hanging-drop vapor diffusion method at room temperature by mixing 2 μl protein solution and 2 μl reservoir solution containing 0.1 M Hepes pH 7.5, 0.2 M Li_2_SO_4_, 0.1 M NaOAc and 25% (w/v) PEG 4000. The reservoir solutions supplemented with 10 to 20% glycerol were used for cyroprotection of the complex crystals. The crystals were then flash-frozen in liquid nitrogen and stored for synchrotron-radiation data collection. The diffraction data were processed using the HKL2000 program suite [19] with data statistics as summarized in Table 2.

**Table 2:** Data collection and refinement statistics.

	hGal1–LN1	hGal3-CRD–LN1	hGal7–LN1
**Data collection**			
Space group	*P* 2_1_2_1_2_1_	*P* 2_1_2_1_2_1_	*P* 2_1_2_1_2_1_
Cell dimensions			
*a*, *b*, *c* (Å)	43.3, 58.2, 111.4	37.1, 57.6, 63.2	30.1, 55.4, 136.5
*α*, *β*, *γ* (°)	90, 90, 90	90, 90, 90	90, 90, 90
Resolution (Å)	30–1.93 (2–1.93)^1^	30–2.208 (2.29–2.208)	30–2.23 (2.31–2.23)
I/s	29.67 (7.11)	38.35 (18.23)	14.59 (6.21)
Completeness (%)	99.5 (96.4)	99.47 (95.45)	98.67 (96.45)
Redundancy	6.7 (6.3)	7.7 (7.8)	4.8 (4.7)
*R*_*sym*_ ^*2*^	0.056 (0.246)	0.051 (0.132)	0.050 (0.277)
**Refinement**			
*R*_*work*_ ^*3*^ */ R*_*free*_ ^*4*^	0.196 / 0.235	0.165 / 0.203	0.196 / 0.225
*B*-factors (mean)	34.40	32.00	30.00
r.m.s. deviations			
Bond lengths (Å)	0.010	0.008	0.007
Bond angles (°)	1.24	1.03	0.85
Ramachandran (%)			
Favored (%)	97	99	98
Outliers (%)	0	0	0
**PDB ID code**	4XBL	4XBN	4XBQ

^1^Statistics for data from the highest-resolution shell are shown in parentheses.

^2^ $R_{sym}=\left(\Sigma \Sigma \left|I_{hkl}-\langle I\rangle \right|\right)\;/\;\left(\Sigma I_{hkl}\right)$, where the average intensity <*I*> is taken overall symmetry equivalent measurements and *I*_*hkl*_ is the measured intensity for any given reflection.

^3^ $R_{work}=\left(\Sigma \left|\left|F_{o}\right|-k\left|F_{c}\right|\right|\right)\;/\;\left(\Sigma \left|F_{o}\right|\right)$, where *F*_*o*_ and *F*_*c*_ are the observed and calculated structure factor amplitudes, respectively.

^4^ *R*_*free*_ was calculated for R factor using only an unrefined subset of reflections data (5%).

### Determination and refinement of the crystal structures

The crystal structures of all complexes were solved by molecular replacement with the PHENIX AutoMR [20] using previously published ligand-free galectin structures as the starting search models: PDB entries 1W6N [14], 2NMN [21] and 1BKZ [16] for hGal1, 3-CRD and 7, respectively. Model building was performed with PHENIX AutoBuild [20]. The resulting electron density maps were of good quality and show clearly the densities belonging to the bound LN1 molecules. The structure of LN1 molecule was created by JLigand version 1.0.35 in CCP4 software suite [22] and built into the density by using Coot [23]. Structures then underwent rounds of manual model rebuilding and refinement with Coot and PHENIX. Detailed refinement parameters are listed in Table 2. The figures were generated in Pymol [24].

## Results and Discussion

### Binding affinity and preference of hGal1, hGal3 and hGal7 for Galβ 1-3/4GlcNAc

We first quantitated the LN1- and LN2-binding affinity of hGal1, 3 and 7 by two different methods, Biolayer interferometry [25] and FP-based competition assays [17, 18]. The resulting *K*_*d*_ values are summarized in Table 1 to show the binding affinity of 3 human galectins with LN1 and LN2. Although the *K*_*d*_ values obtained by Biolayer interferometry (*K*_*d*_^*BI*^) are generally about 2 orders of magnitude greater than those by FP-based competition assays (*K*_*d*_^*FP*^), both measurements reveal a highly consistent trend. hGal7 showed significantly increased affinity for LN1 (*K*_*d*_^*BI*^ = 270 μM or *K*_*d*_^*FP*^ = 6.6 μM) than for LN2 (*K*_*d*_^*BI*^ = 410 μM or *K*_*d*_^*FP*^ = 19.7 μM), whereas hGal1 and hGal3 are more specific for LN2 (Table 1). Biolayer interferometry was known to measure the binding affinity and additional kinetic detail of the given compound/protein complex, such as kinetic constants of association and dissociation [26]. Recently Biolayer interferometry was applied to characterize the binding properties of galectins [27]. It is common that different methods measuring the same binding event often produce different *K*_*d*_ values. The difference could vary from 1 to 2 orders of magnitude [12, 28–30], but the most important issue is to see if there is a consistent trend on the binding when comparing diffrerent methods. Overall, the LN1/LN2 binding preference of galectins-1, -3 and -7 are not strict. For instance, only a maximum 3-fold difference in LN1 and LN2 binding affinity of hGal7 was observed in this study, suggesting the possibility of functional redundancy among members of galectin family [31, 32]. Given the feature of multivalent interactions in the context of galectin/glycan lattices, even 2- or 3-fold change in their individual interactions would result in substantially higher activity and/or a more dramatic effect [33]. As a matter of fact, several studies also correlate the binding preference of LN1 or LN2 with physiological activities [34]. Structural information is thus necessary to delineate the insight at molecular level.

### Overall structures of hGal1, 3-CRD and 7 in complex with LN1

**Fig 1:**
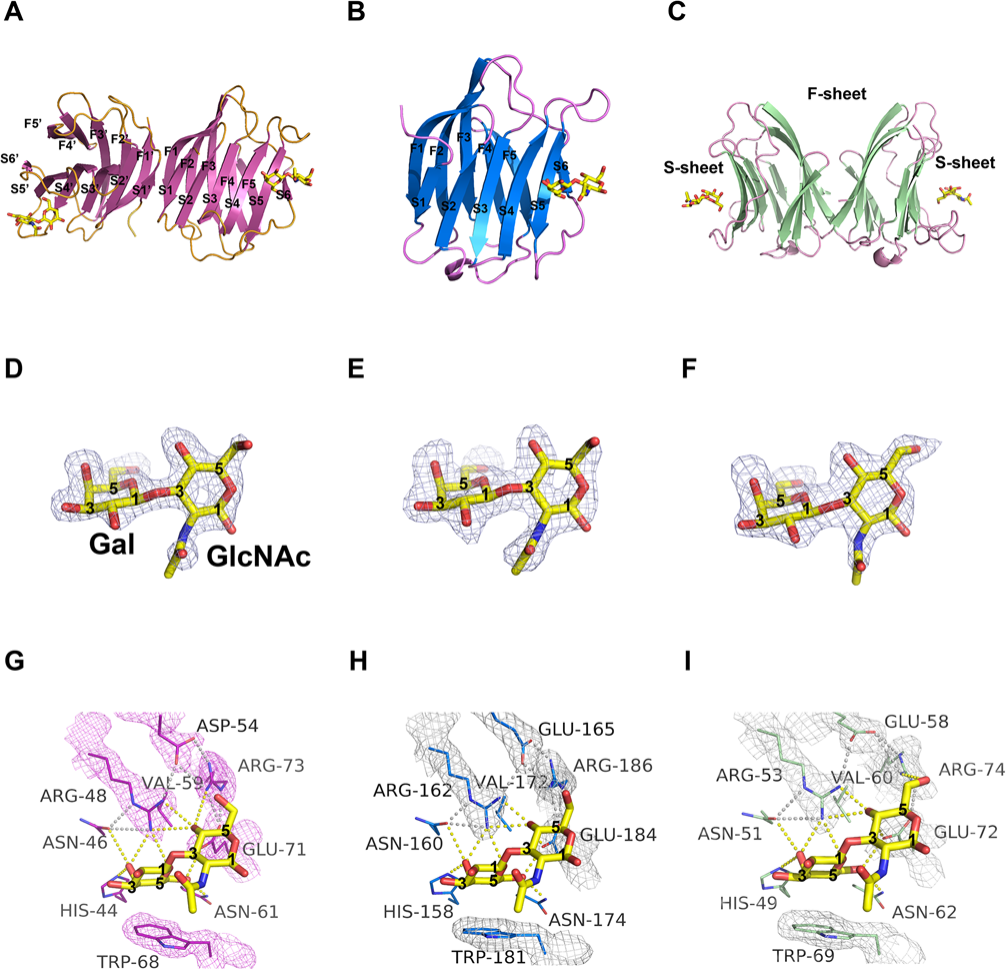
Structural overview of hGal1, 3-CRD and 7 in complex with LN1. (A-C) Ribbon representations of three LN1-hGal complexes where LN1 (shown in yellow stick model) is bound to hGal1 (A), hGal3-CRD (B) and hGal7 (C). Numbering of the β-strands of S-sheet (S1-S6) and F-sheet (F1-F5) is also shown as indicated. (D-F) *F*_*o*_ *-F*_*c*_ omit electron density map of LN1 (contoured at 2.5σ) bound to hGal1 (D), hGal3-CRD (E) and hGal7 (F). To make it clear, carbons 1, 3 and 5 of Gal and GlcNAc are labeled. (G-I) β-galactoside-recognition site of hGal1-LN1 (G), hGal3-CRD-LN1 (H) and hGal7-LN1 (I) complexes. Residues involved in LN1 recognition are highlighted with 2*F*_*o*_ *-F*_*c*_ electron density (contoured at 1σ). Polar interactions among galectin residues and within galectin/LN1 complex are shown as gray and yellow dash lines, respectively.

hGal1, the CRD of hGal3 (hGal3-CRD) and hGal7 in complex with LN1 were crystallized by either a soaking or co-crystallization method. Their crystal structures were then determined between 1.9 and 2.2 Å by molecular replacement on the basis of the published ligand-free structures (PDB IDs: 1W6N [14], 2NMN [21] and 1BKZ [16] corresponding to hGal1, hGal3-CRD and hGal7, respectively) as the starting search models. Statistics of data processing and refinement parameters of the structures are summarized in Table 2. While only one monomer exists in the asymmetric unit of hGal3-CRD co-crystal, each asymmetric unit of hGal1 and hGal7 harbors a distinct symmetric dimer (Fig 1A–1C). Specifically, the dimer of hGal1 exists in a side-by-side manner, whereas hGal7 dimer is present in a back-to-back arrangement. Because the two protomers of the hGal1 and hGal7 dimers are almost identical to each other, we refer to chain A of each crystal structure in the following discussion. All the observed CRDs adopt a typical galectin fold which is composed of two antiparallel β-sheets of six (S1-S6) and five (F1-F5) strands, jointly forming a β-sheet sandwich structure and therefore named as S-sheets and F-sheets, respectively. The S1-S6 β-strands constitute a concave surface to which β-galactoside-containing glycans are bound. Electron density belonging to the bound LN1 is clearly identified in the *Fo-Fc* electron density map (Fig 1D–1F), indicating that the LN1 molecule in the complex is well ordered and all sugar rings in the LN1 adopt a chair conformation. Of note, overall root mean square deviation (RMSD) values among the newly determined LN1-bound galectins (hGal1, hGal3-CRD and hGal7), the ligand-free and the LN2-bound galectins (PDB IDs: 1W6N, 3ZSM [35] and 1BKZ for ligand-free hGal1, hGal3-CRD and hGal7; PDB IDs: 1W6P [14], 1KJL [15] and 5GAL [16] for LN2-loaded hGal1, hGal3-CRD and hGal7) are quite small. The values of RMSD (C^α^ atoms) between ligand-free and LN1-bound hGal1, 3 and 7 are of 0.57, 0.20 and 0.53 Å, respectively. RMSD values (C^α^ atoms) of 0.10, 0.18 and 0.59 Å are presented between ligand-free and LN2-bound hGal1, hGal3 and hGal7 structures, respectively. Therefore, any differences due to the glycosidic linkages (β1–3 or β1–4) in LN1 and LN2 appear not to seriously distort the overall structure of hGal1, hGal3-CRD and hGal7.

Generally the galactose moiety (GAL) forms more H-bonds with the amino acid residues in the CRD than the N-acetylglucosamine moiety (GlcNAc), supporting the idea that GAL serves as a major recognition component. The GAL of LN1 interacts with a series of conserved residues located on S4-S6 β-strands and the loop connecting S4 and S5 strands, which include His44^hGal1^/158^hGal3^/49^hGal7^, Asn46^hGal1^/160^hGal3^/51^hGal1^, Arg48^hGal1^/162^hGal3^/53^hGal7^, Asn61^hGal1^/174^hGal3^/62^hGal7^ through hydrogen bond (H-bond) networks, and Trp68^hGal1^/181^hGal3^/69^hGal7^ via van der Waals contacts (Fig 1G–1I). In particular, the conserved Arg48^hGal1^/162^hGal3^/53^hGal7^ residues play an important role in mediating interactions between hGal1, hGal3, hGal7 and their corresponding LN1 molecules, respectively. Specifically, the Arg48^hGal1^/162^hGal3^/53^hGal7^ residues not only bridge H-bonds to several oxygen atoms of LN1 including C4-OH, O5 of GAL and C4-OH of GlcNAc, but also connect peripheral carbohydrate-interacting amino acid residues such as Asn46^hGal1^/160^hGal3^/51^hGal7^, Asp54^hGal1^/Glu165^hGal3^/58^hGal7^, and Arg73^hGal1^/186^hGal3^/74^hGal7^ to form a characteristic interacting network of H-bonds and electrostatic interactions which are optimal for carbohydrate orientation in the binding curvature (Fig 1G–1I) [16, 36]. The density maps of the LN1-interacting amino acid residues are shown in a satisfying quality to define the environment of the LN1-binding site.

### Structural basis for LN1- and LN2-binding preferences of hGal1, hGal3-CRD and hGal7

In accordance with the X-ray crystal structures, the numbers and distances of specific H-bond interactions involved in the recognition of LN1/ LN2 by hGal1, hGal3 and hGal7 are measured and summarized in Table 3 as the basis to interpret their distinct binding specificity. Generally, hGal1 and hGal3 appear to have shorter distances (in average) in H-bonds to both GAL and GlcNAc moieties of LN2 than those of LN1. In contrast, hGal7 has more H-bonds to GAL moiety and a characteristic shorter distance with GlcNAc in LN1, as compared to those in LN2. These results correlate well with the aforementioned difference in the binding affinity. The static X-ray structures may not always correspond to the behavior of proteins in solution, further studies such as NMR or MD simulations based on these LN1/LN2-galectin complex structures would offer more insights with their binding dynamics in solution.

**Table 3:** Comparison of polar interactions between human Galectins and LN1/2^a^.

		hGal1-LN1		hGal1-LN2^b^	hGal3-CRD-LN1	hGal3CRD-LN2^b^	hGal7-LN1		hGal7-LN2^b^
Sugar atom	Amino acid atom in hGal1/3/7	Mol.A	Mol.B	(1W6P_Mol.A)	Mol.A	(1KJL)	Mol.A	Mol.B	(5GAL_Mol.B)
GlcNAc O3	Glu 71/184/72 O_ε_1	-		3.33	-	-	-		3.15
GlcNAc O3	Glu 71/184/72 O_ε_2	-		2.59	-	2.54	-		2.62
GlcNAc O3	Arg 48/162/53 Nη1	-		2.73	-	2.77	-		3.07
GlcNAc O3	Arg 48/162/53 Nη2	-		2.99	-	3.15	-		3.42
GlcNAc O4	Glu 71/184/72 O_ε_1	3.02	3.25	-	-	-	3.43		-
GlcNAc O4	Glu 71/184/72 O_ε_2	2.65	2.40	-	2.58	-	2.48	2.87	-
GlcNAc O4	Arg 48/162/53 Nη1	2.88	2.90	-	2.78	-	2.77	2.54	-
GlcNAc O4	Arg 48/162/53 Nη2	3.22	3.21	-	3.08	-	2.92	3.07	-
GlcNAc O4	Arg 73/186/74 Nη2	3.31	3.47	-	-	-	-		-
GlcNAc N2	Water 23/01/-	-		2.80	-	2.9	-		-
Avg. interaction distance with GlcNAc		3.03		2.89	2.81	2.84	2.87		3.07
Gal O4	His 44/158/49 N_ε_2	2.87	2.71	2.74	2.76	2.8	2.89	2.9	2.95
Gal O4	Asn 46/160/51 O_δ_1	3.37	3.23	3.15	3.26	3.13	3.24	3.25	2.87
Gal O4	Arg 48/162/53 Nη2	2.77	3.02	3.01	2.93	2.86	2.91	2.72	2.6
Gal O5	Arg 48/162/53 Nη2	3.0	3.1	2.88	3.0	2.96	2.94	2.94	2.81
Gal O5	Glu 71/184/72 O_ε_2	3.37	3.06	3.12	3.31	3.3	3.15	3.28	-
Gal O6	Asn 61/174/62 N_δ_2	2.89	2.75	2.88	2.89	2.74	2.75	2.8	2.63
Gal O6	Glu 71/184/72 O_ε_1	-		-	-	-	-		2.85
Gal O6	Glu 71/184/72 O_ε_2	2.7	2.78	2.67	2.72	2.7	2.71	2.5	-
Avg. interaction distance with Gal		2.97		2.92	2.98	2.93	2.93		2.79

^a^hydrogen bond interactions were identified with program, Pymol. Values shown are distances in Å.

^b^LN2-complexed hGal1/3-CRD/7 structures reported in previous literatures were used for comparison.

**Fig 2:**
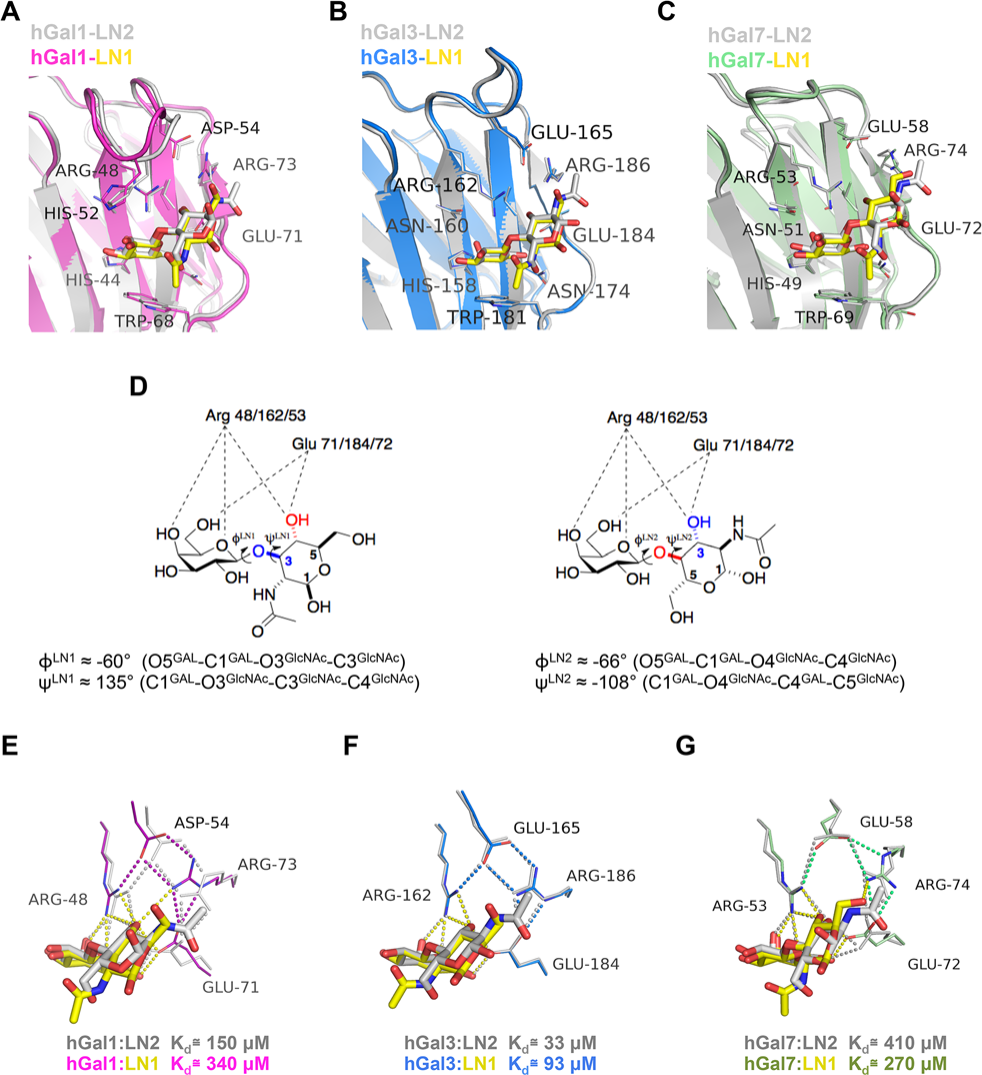
Pairwise comparison of the β-galactoside-recognition site in the LN1-hGal1/3-CRD/7 and LN2-hGal1/3-CRD/7 complexes. (A-C) Structures of hGal1 (PDB ID: 1W6P), hGal3-CRD (1KJL) and hGal7 (5GAL) in complex with LN2 (all shown in gray) were superimposed, respectively, with the LN1 (in yellow)-containing structures of hGal1 (pink), hGal3-CRD (cyan) and hGal7 (green). (D) Diagrams delineate the different interaction geometries of LN1 (left) and LN2 (right) with respect to hGal1/3-CRD/7. Definition and values of the glycosidic torsion angles (ϕ and ψ) for LN1 and LN2 molecules are also listed. (E-G) Close-up view of the unique salt bridge networks in hGal1 (E), hGal3-CRD (F) and hGal7 (G), with a superposition of their LN2 and LN1-complex structures. Polar interactions among LN2-complexed structures are shown in gray dash lines while the interactions among amino acid residues in LN1-loaded structures are indicated by colored dash lines according to hGal1 (pink), 3-CRD (blue) and 7 (green), respectively, and all interactions related to galectin/LN1 complexes formation are colored in yellow.

Overall, the LN1-recognition modes of hGal1, hGal3-CRD and hGal7 are quite similar to those observed in the LN2-bound complex structures. Structural superimpositions of LN2-complexed hGal1, hGal3-CRD and hGal7 structures (PDB IDs: 1W6P, 1KJL and 5GAL, respectively) with their LN1-loaded ones (Fig 2A–2C) indicate that the GAL moiety of LN1 and LN2 is overlapped well. Even though the GlcNAc moiety of LN1 and LN2 interacts with the same amino acid residues, the GlcNAc moiety was found to adopt a different orientation in LN1- and LN2-complex structures. Specifically, the average torsional angles ϕ^LN1^ (-60°, defined by O5^GAL^–C1^GAL^–O3^GlcNAc^–C3^GlcNAc^ of LN1) and ϕ^LN2^ (-66°, defined by O5^GAL^–C1^GAL^–O4^GlcNAc^–C4^GlcNAc^ of LN2) that depict the position of GAL relative to the glycosidic bond are quite similar (Fig 2D). On the other hand, the torsional angle ψ is to characterize the orientation of GlcNAc relative to the glycosidic bond. The average ψ^LN1^ (135°, defined by C1^GAL^–O3^GlcNAc^–C3^GlcNAc^–C4^GlcNAc^ of LN1) is dramatically different from the average ψ^LN2^ (-108°, defined by C1^GAL^–O4^GlcNAc^–C4^GlcNAc^–C5^GlcNAc^ of LN2). This ~240° shift allows the three important OH groups of galectin-bound LN1 (C4-OH and C6-OH of Gal and C4-OH of GlcNAc) to form the binding interactions not only essential for the gelectin recognition, but also homologous to those produced by the OH groups of galectin-bound LN2 (C4-OH and C6-OH of Gal and C3-OH of GlcNAc) (Fig 2D) [10, 37, 38]. The glycosidic torsional angles in this study were defined in previous reports [16, 36]. Notably the C4-OH group of GlcNAc in LN1 correlates with C3-OH group of GlcNAc in LN2 so that they are located at the equivalent position to form pivotal H-bonds with Arg48^hGal1^/162^hGal3^/53^hGal7^ and Glu71^hGal1^/184^hGal3^/72^hGal7^ that are highly conserved among human galectins (Fig 2D).

### Roles of Asp54^hGal1^/Glu165^hGal3^/58^hGal7^ in mediating a unique salt bridge network

Furthermore, we found several subtle but major differences in the carbohydrate-binding sites of hGal1, hGal3-CRD and hGal7 (Fig 2E–2G). First, hGal1 and 7 have the electrostatic network consisting of Arg48^hGal1^/53^hGal7^, Asp54^hGal1^/Glu58^hGal7^, Glu71^hGal1^/72^hGal7^ and Arg73^hGal1^/74^hGal7^ [16, 36]. These residues adopt dissimilar conformations and orientations when interacting with LN1 or LN2 molecules (Fig 2E and 2G), while the corresponding network of hGal3-CRD (Arg162, Glu165, Glu184 and Arg186) interacts either with LN1 or LN2 in almost the same manner (Fig 2F). Neither of Asp54^hGal1^, Glu165^hGal3^ and Glu58^hGal7^ interacts with the disaccharides directly, but the way they interact with the Arg counterparts forming the salt bridge appears to be dissimilar. The unique salt-bridge network mediated by Glu58^hGal7^ (Fig 2G) was different from those mediated by the corresponding residues Asp54^hGal1^, Glu165^hGal3^ (Fig 2E and 2F). According to classification to define the salt bridge geometry [39], Glu58^hGal7^ forms two weaker monodentate N-O bridges with Arg53^hGal7^ and Arg74^hGal7^ (the important LNs-interacting residues Arg53 and Glu72) to mediate the salt bridge network (Fig 2G), while both monodentate (Arg48^hGal1^–Asp54^hGal1^ and Arg162^hGal3^–Glu165^hGal3^) and bidentate (Asp54^hGal1^–Arg73^hGal1^ and Glu165^hGal3^–Arg186^hGal3^) interactions are observed in hGal1 and 3 (Fig 2E and 2F, respectively). Except for the residues involved in the unique salt-bridge network, especially those in hGal1 (Asp54^hGal1^–Glu71^hGal1^–Arg73^hGal1^) and hGal7 (Glu58^hGal7^–Glu72^hGal7^–Arg74^hGal7^) characteristic of larger RMSD (all atoms) (0.98 and 0.64Å, respectively, see Fig 2E–2G), the majority of LN1/2-contact residues of hGal1, 3 and 7, such as His44^hGal1^/158^hGal3^/49^hGal7^, Asn46^hGal1^/160^hGal3^/51^hGal7^, Arg48^hGal1^/162^hGal3^/53^hGal7^, Val59^hGal1^/172^hGal3^/60^hGal7^, Asn61^hGal1^/174^hGal3^/62^hGal7^ and Trp68^hGal1^/181^hGal3^/69^hGal7^ are highly structural conserved with much smaller RMSD (all atoms) of 0.25, 0.13 and 0.29 Å, respectively (Fig 2A–2C).

**Fig 3:**
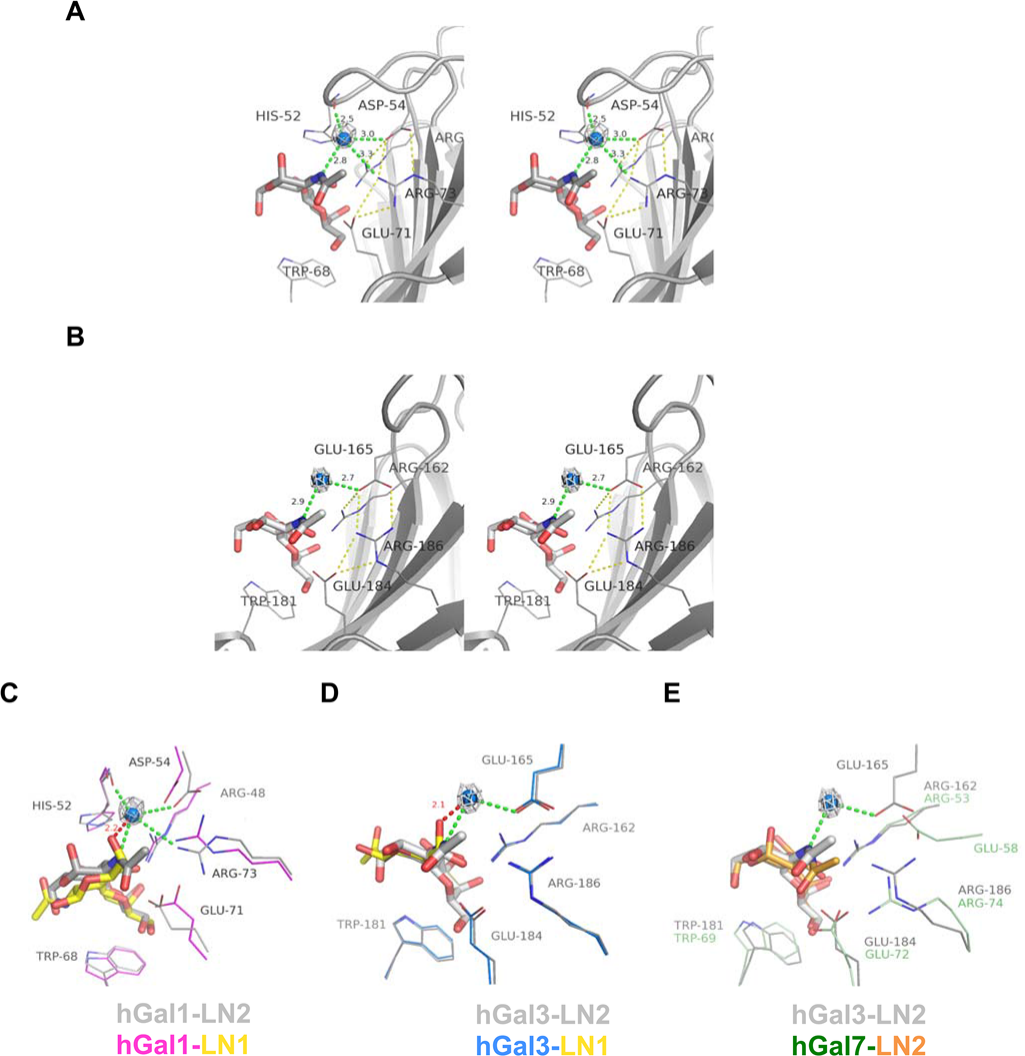
Water-mediated interactions of hGal1 and hGal3 with the LN2 molecules. (A and B) Stereoview of LN2 molecule bound in the carbohydrate-recognition site of hGal1 (PDB ID: 1W6P) and hGal3-CRD (1KJL), respectively. LN2 ligand is depicted as gray stick model. The water (blue sphere) is coordinated by the H-bonds (green dashes) from N2 atom of LN2 and amino acid residues of galectins. *2F*_*o*_ *-F*_*c*_ omit electron density (gray mesh) of the water molecules are highlighted and contoured at 1σ. Unique salt bridge network of hGal1 and hGal3-CRD are indicated as yellow dashes. (C and D) Structural superposition of LN1 and LN2 complex structures from hGal1 (C) and hGal3-CRD (D). The C5-hydroxyl group of LN1 (yellow sticks) in hGal1 and hGal3-CRD complexes makes close contact with the coordinated water in LN2-hGal1 andLN2-hGal3-CRD complex with a distance of 2.2 and 2.1 Å, respectively. (E) Structure of hGal7 (shown in color green) in complex with LN2 (orange) is superimposed with LN2-hGal3-CRD complex structure (all in gray). Most LN2-contacting residues in hGal7 (such as Arg53^hGal7^, Trp69^hGal7^, Glu72^hGal7^ and Arg74^hGal7^) are well superimposed with those of hGal3, except the Glu58^hGal7^ residue. Location of Glu58^hGal7^ is distinctive from Glu165^hGal3^/ Asp54^hGal1^ and distance between Glu58^hGal7^ and N2 atom of LN2 is too far for them to coordinate a water molecule in between.

Moreover, water-mediated interactions, identified to exist in the Asp54^hGal1^, Arg73^hGal1^ and Glu165^hGal3^-situated salt-bridge networks (Fig 3A and 3B), were proposed to correlate with the LN2-binding specificity. A water was hold by Asp54^hGal1^, Arg73^hGal1^ and the N2 atom of GlcNAc in the hGal1/LN2 complex via formation of H-bonds (Fig 3A), whereas Glu165^hGal3^ and the N2 of GlcNAc were observed to clamp a water molecule in the hGal3/LN2 complex (Fig 3B). The similar interactions were also found in the hGal1- and hGal3-lactose complexes (PDB IDs: 1W6O [14] and 2NN8 [21]) where the O2 atom of glucose was comparable to the N2 of GlcNAc in the previous two structures. However, there are no such water-mediated interactions in our hGal1-LN1 and hGal3-LN1 structures. As aforementioned, N-acetyl group in LN2 is structurally equivalent to C5-hydroxymethyl group in LN1 (Fig 2D). To accommodate an extra water molecule becomes impossible owing to the limited space for C5-hydroxymethyl group of LN1 and the residues participating in the salt-bridge network (Fig 3C and 3D). Meanwhile, neither of our hGal7-LN1 and the hGal7-LN2 complexes (PDB ID: 5GAL) [16] contains the aforementioned water-mediated interactions (Fig 3E). The main cause is the location of Glu58^hGal7^ (Fig 3E) that is distinctive from that of Asp54^hGal1^ and Glu165^hGal3^. Apparently Glu58^hGal7^ was relatively remote from the bound sugar in hGal7, as compared to the analogous Asp54 in hGal1 and Glu165 in hGal3.

**Fig 4:**
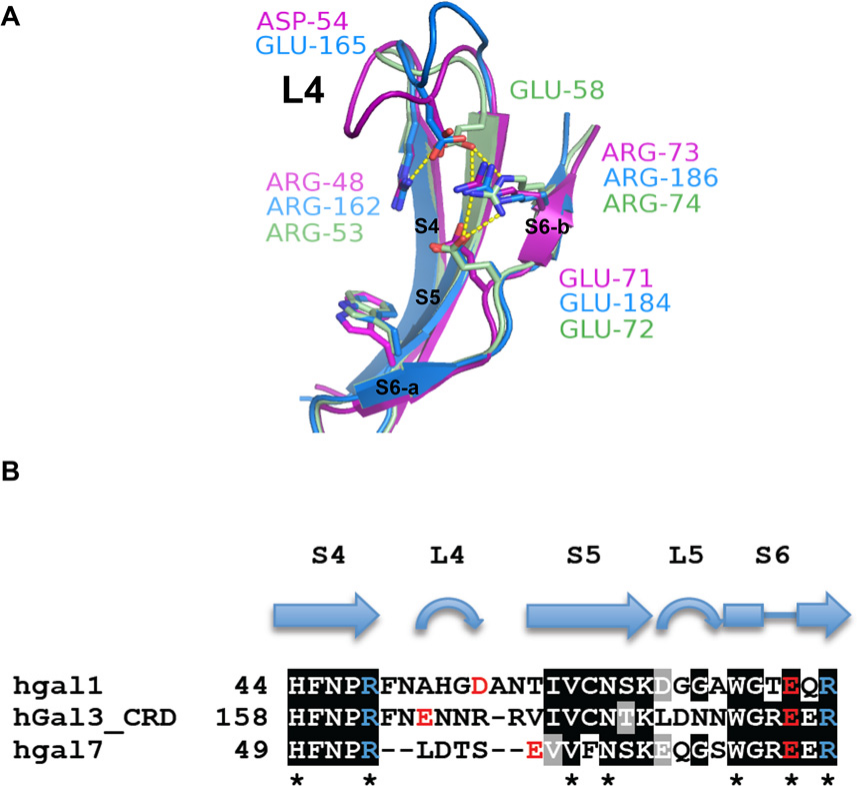
Structural comparisons among hGal1, 3-CRD and 7. (A) S4-S6 β-strands of hGal1 (pink), 3-CRD (blue) and 7 (green) are superimposed. The unique salt bridge network of hGal1 (R48-D54-E71-R73), 3-CRD (R162-E165-E184-R186) and 7 (R53-E58-E72-R74) are shown in stick models. (B) Structure-based sequence alignment of S4-S6 β-strands of hGal1, 3-CRD and 7. Secondary structures were designated according to the resolved x-ray structures. The highly conserved LNs-interacting residues among hGal1, 3-CRD and 7 are indicated by asterisks. Residues involved in unique salt bridge network of hGal1, 3-CRD and 7 are colored in either red (Glu/Asp) or blue (Arg).

Superimposition of LN1-complexed hGal1, 3 and 7 indicated an important difference in the loop (denoted as L4) between S4 and S5 β-strands (Fig 4), and that Asp54^hGal1^, Glu165^hGal3^ and Glu58^hGal7^ are differently positioned in L4. L4 of hGal7 is shorter than the counterpart in hGal1 and hGal3 (Fig 4B). The additional amino acid residues in the L4 of hGal1 and 3 thus throng round the space in the vicinity of their carbohydrate-binding sites (Fig 4A). Unlike Asp54^hGal1^ and Glu165^hGal3^ both situated in the internal of L4, Glu58^hGal7^ is resided in either the end of L4 or the beginning of S5 β-strand (Fig 4A and 4B), leading to a differently oriented salt bridge network from those of hGal1 and hGal3 (Fig 2E–2G). Such an arrangement makes it impossible for Glu58^hGal7^ to coordinate with the N2 atom of LN2 for additional water-mediated interactions (Fig 3E). Taken together, the LN2-binding preference is possibly linked to the presence of water-mediated interactions, which is under the control of the properly positioned salt-bridge in L4.

Even Asp54^hGal1^ and Glu165^hGal3^ have a dissimilar location in L4 (Fig 4B), i.e. Asp54^hGal1^ is resided at the end of the β-turn structure in L4, while Glu165^hGal3^ appears as the first residue of the β-turn. Since Asp54^hGal1^ and Glu165^hGal3^ residues mediate ionic interactions to their adjacent Arg residues, apparently the location of Asp54^hGal1^ and Glu165^hGal3^ results in the distinct conformation of L4 in hGal1 and hGal3-CRD (Fig 4A). This explains the reason why hGal3, but not hGal1, binds with tumor-related TF antigen (Galβ1-3GalNAc) [40]. When the x-ray structure of hGal3-TF antigen is superimposed with the hGal3-LN1 structure, the galactose moiety is roughly overlapped (ϕ = -90° vs. -65°, respectively), but the adjacent sugar residue adopts a very different orientation (ψ = 75° vs. 136°). The observation again demonstrates that, in addition to the primary interactions with the galactose residue, the neighboring sugar has to rotate the glycosidic bond to interact well with several important residues, such as Arg162^hGal3^ and Glu184^hGal3^. Although Glu165^hGal3^ in L4 is not involved in the binding, this residue forms the salt-bridge network to connect with Arg162^hGal3^ and Glu184^hGal3^.

Salt bridge motifs are known to play a key role in a number of functions, such as stabilization of protein folds, and arrangement of key residues or waters for the purpose of catalysis or molecular recognition [39, 41–43]. In this work the unique salt bridge motifs mediated by Asp54^hGal1^, Glu165^hGal3^ and Glu58^hGal7^ were identified to coordinate with the vicinal carbohydrate-binding sites to distinguish the bound sugar ligands, which accounts for the LN1/2-binding preferences of hGal1, 3 and 7, respectively.

**Fig 5:**
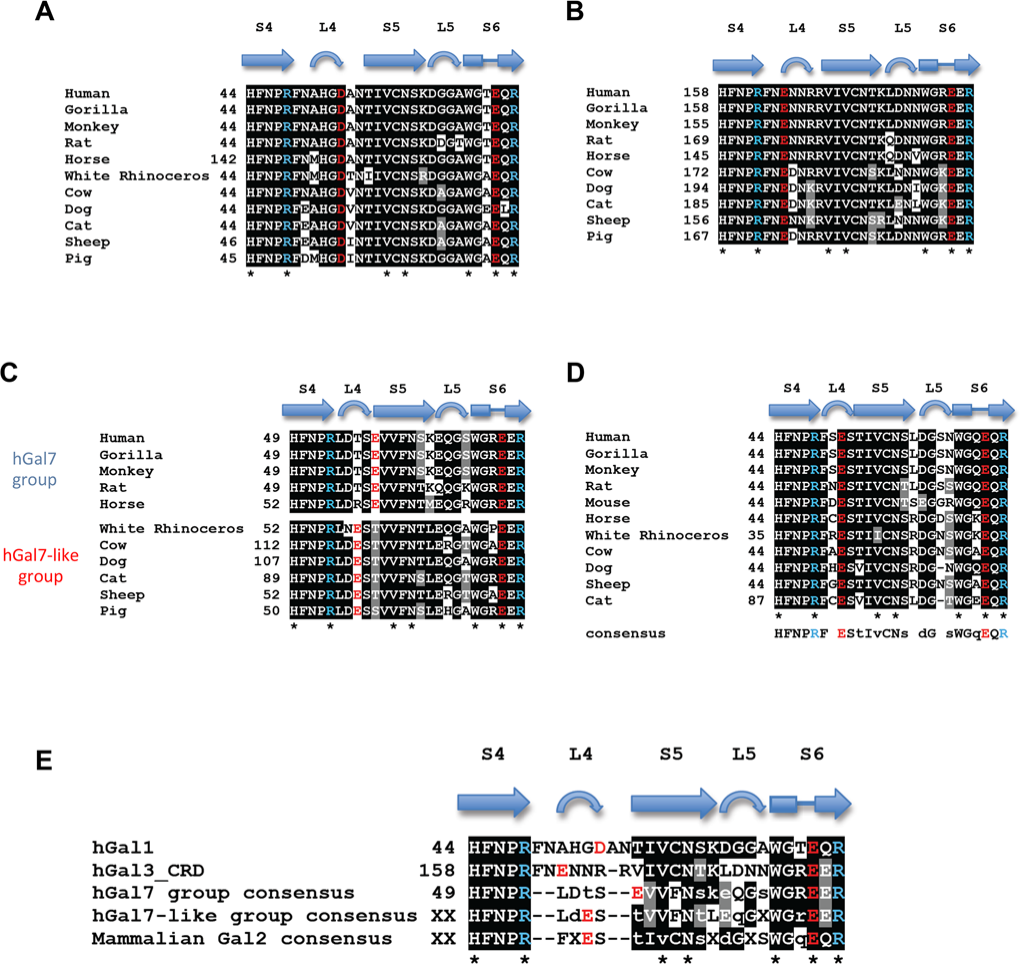
Conservation of the L4 regions among mammalian galectins. Structure-based sequence alignment of S4-S6 β-strands of mammalian galectin-1s (A), galectin-3s (B), galectin-7s (C) and galectin-2s (D). According to the variation at corresponding position of Glu58^hGal7^, mammalian galectin-7s are further divided into two subgroups, the hGal7 group and hGal7-llike group. Sequence comparison of L4 regions of human Galectin-1, 3 and mammalian Galectin-2, 7 (E). Invariant LNs-contacting residues of galectins are indicated by asterisks. Residues involved in unique salt-bridge network are colored according to negative (red) or positive (blue) charged properties. The sequences of mammalian galectins were selected from human (*Homo sapiens*: galectin-1 [NP_002296.1], galectin-2 [NP_006489.1], galectin-3 [BAA22164.1] and galectin-7 [NP_002298.1]), Gorilla (*Gorilla gorilla gorilla*: galectin-1 [XP_004063482.1], galectin-2 [XP_004063485.1], galectin-3 [XP_004055252.1] and galectin-7 [XP_004060726.1]), Monkey (*Macaca mulatta*: galectin-1 [NP_001162098.1], galectin-2 [XP_001087063.1], galectin-3 [NP_001253292.1] and galectin-7 [NP_001083444.1]), Rat (*Rattus norvegicus*: galectin-1 [NP_063969.1], galectin-2 [NP_598283.1], galectin-3 [NP_114020.1] and galectin-7 [NP_072104.2]), Horse (*Equus caballus*: galectin-1 [XP_001501082.2], galectin-2 [XP_001499566.2], galectin-3 [XP_005605252.1] and galectin-7 [XP_001496714.2]), Rhinoceros (*Ceratotherium simum simum*: galectin-1 [XP_004418181.1], galectin-2 [XP_004418176.1] and galectin-7 [XP_004441551.1]), Cow (*Bos taurus*: galectin-1 [NP_786976.1], galectin-2 [NP_001244020.1], galectin-3 [NP_001095811.1] and galectin-7 [XP_002695023.2]), Dog (*Canis lupus familiaris*: galectin-1 [ADR80617.1], galectin-2 [NP_001271396.1], galectin-3 [NP_001183972.1] and galectin-7 [NP_001183972.1]), Cat (*Felis catus*: galectin-1 [XP_003989294.1], galectin-2 [XP_006934157.1], galectin-3 [XP_003987704.1] and galectin-7 [XP_003987704.1]), Sheep (*Ovis aries*: galectin-1 [AAT38511.1], galectin-2 [XP_004007664.1], galectin-3 [XP_004010713.1] and galectin-7 [XP_004010713.1]), Pig (*Sus scrofa*: galectin-1 [NP_001001867.1], galectin-3 [NP_001090970.1] and galectin-7 [NP_001136315.1]) and Mouse (*Mus Musculus*: galectin-2 [NP_079898.2]).

As discussed previously, the sequence alignment of hGal1, 3 and 7 indicates that L4 is highly variable. But L4 of each galectin appears to be highly conserved among mammalian species (Fig 5), suggesting that L4 is likely to be evolved into different structures. Each of them is well arranged by heavy H-bond connections between carbonyl and amide groups on their polypeptide backbones, supporting the previous hypothesis that different architectures and dynamics of these variable L4 regions might be functionally relevant for the carbohydrate-binding specificities and thus influence the biological properties of each galectin member [16, 40, 44–46]. Of particular note, the unique salt bridge networks of hGal1 (Arg48^hGal1^-Asp54^hGal1^-Glu71^hGal1^-Arg73^hGal1^) and hGal3 (Arg162^hGal3^-Glu165^hGal3^-Glu184^hGal3^-Arg186^hGal3^) are highly conserved (Fig 5A and 5B), while those in Gal7s are divided into two divergent groups due to the variations in the position of Glu58^hGal7^ (Fig 5C). One group of mammalian Gal7s has the corresponding Glu in the end of L4 (hGal7 group), while the other group has this Glu shifted into the middle of L4 (hGal7-like group). Whether the hGal7-like group has a similar LN1-binding preference requires further investigations.

Moreover, it was reported that Gal1 and Gal2 are two most closely related members in the prototype galectin subfamily, and they share up to 43% amino acid sequence identity, in comparison with the 32% sequence identity between hGal1 and hGal7 [44, 46, 47]. In accordance with the analysis of Hirabayashi *et al*., both rat galectin-2 and hGal7 displays prominent LN1-preferred binding activity [12]. Multiple sequence alignment of mammalian Gal2s revealed that they are highly conserved (Fig 5D) and characterized with a shorter L4 sequence that is reminiscent of hGal7-like group (Fig 5E). These analyses strongly suggested that the variable L4 region of galectins is highly relevant to the LN1- or LN2-preferred binding activity. To further demonstrate such relationship, alanine mutation was introduced to replace E165 or R186 of hGal3. Although the binding affinity of hGal3-E165A for LN1 and LN2 was reduced 2.5- and 8.5-fold, respectively (Table 1), the ratios of *K*_*d*_^*LN1*^/ *K*_*d*_^*LN2*^ were changed from 0.35 of wild-type hGal3 to 1.22 of hGal3-E165A (Table 4), suggesting that the original LN2-preferred binding of hGal3 was shifted to LN1-preference. Obviously the L4-directing Asp/Glu residue is indispensible for affecting LN1/2 binding preference. By mediating distinct salt-bridge network and water-mediated interaction, these charged residues at L4 serve as a key structural element to fine-tune the carbohydrate recognition. Nevertheless, the other site-directed mutant hGal3-R186A was found to lose its binding ability for both LN1 and LN2 although it was prepared with success and was no structurally deviated from the wild type hGal3. The lost binding was realized owing to the close relationship of Arg186^hGal3^ with Glu184^hGal3^ (a critical LN1/2-contact residue) [34, 48]. Furthermore, Bonzi *et al*. identified a conformational change of Arg73^hGal1^ (corresponding to Arg186^hGal3^ and Arg74^hGal7^) that was induced by the presence of a unique peptide, λ5-UR of pre-B cell receptor (pre-BCR) [49], leading to the modified carbohydrate-binding specificity of hGal1 (e.g. a 3-fold decrease for Lacto-N-neotetraose (LNnT) and a 10-fold increase for α3-SiaLacNAc). The binding change is crucial for pre-B cell maturation [49, 50], suggesting that the distinct salt-bridge network of galectin members are not only relevant to LN1- or LN2-preference, but also critical to determine the binding preference of other ligands.

**Table 4:** LNs-binding preference for hGal1, 3 and 7.

	LN1/LN2 ratio^1^	
Protein	Current study	Previous study^4^
hGal1	0.44^2^ (0.53^3^)	0.79
hGal3	0.35^2^ (0.23^3^)	0.65^5^
hGal7	1.52^2^ (2.97^3^)	2.7
hGal3-E165A	1.22^2^	

^1^To quantitatively evaluate LN1- or LN2-binding preference of hGal1, 3 and 7, LN1/LN2 ratios (*K*_*d*_^*LN1*^ */K*_*d*_^*LN2*^) were calculated based on the *K*_*d*_ values presented in Table 1.

^2^The values were obtained based on the *K*_*d*_^*BI*^ values determined by Biolayer Interferometry.

^3^The values were obtained based on the *K*_*d*_^*FP*^ values determined by FP-based competition assays.

^4^It was reported by Hirabayashi, J. *et al*. [12].

^5^The CRD domain was used instead of full-length hGal3.

## Conclusions

In summary, our structural studies of hGal1, hGal3-CRD and hGal7 explain how galectins exhibit the binding preference for Galβ1-3/4GlcNAc disaccharides. Since the galactose moiety affords primary interactions, the GlcNAc has to adopt different orientations for keeping comparable H-bonds with several Arg and Glu/Asp residues in a salt-bridge network. Because of the Glu/Asp resided in the variable loop L4, the length of L4 and the location of the Glu/Asp are found to influence the geometry of the salt bridge, resulting in the LN1/2-binding preference.

## Supporting Information

S1 Fig Octet Red biolayer interferometry-based equilibrium analysis for LN1 and LN2 binding to tip-bound hGal1, 3 and 7, respectively. Steady state *K*_*d*_ values and statistical parameters of the fitting were listed by fitting curve from biolayer interferometry experiments at 27°C. Experimental procedures were detailed as those described in Materials and Methods.
(TIF)

S2 Fig Fluorescence polarization analysis of (A) hGal1 (0–250 μM), (B) hGal3 (0–230 μM) and (C) hGal7 (0–360 μM) with LN2-FITC (0.1 μM) at 4°C. Duplicate measurements are shown for all data points. The curves represent fitting of the data by nonlinear regression to the simplified formula for a one to one interaction by Prism 5.0 software (GraphPad, San Diego, CA). Their *K*_*d*_ values were extracted from the fitting and shown as indicated, respectively.
(TIF)
