# Supplementary figures and images for: Structural Basis Underlying the Binding Preference of Human Galectins-1, -3 and -7 for Galβ1-3/4GlcNAc
Source: PLoS One. 2015 May 6;10(5):e0125946. doi: 10.1371/journal.pone.0125946 (PMC4422656; doi:10.1371/journal.pone.0125946)

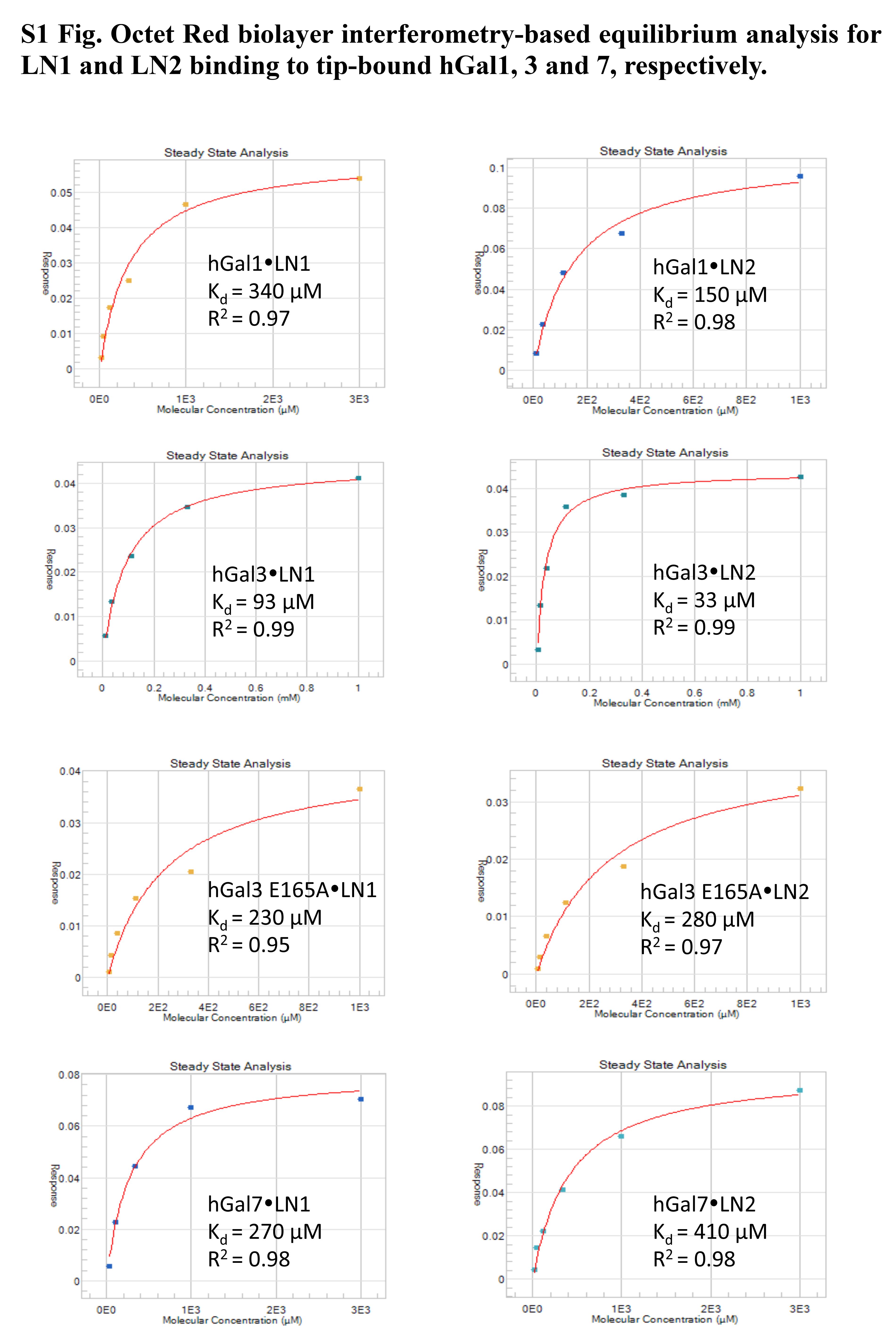

Supplement: S1 Fig — Steady state K d values and statistical parameters of the fitting were listed by fitting curve from biolayer interferometry experiments at 27°C. Experimental procedures were detailed as those described in Materials and Methods. (TIF) [file pone.0125946.s001.tif]

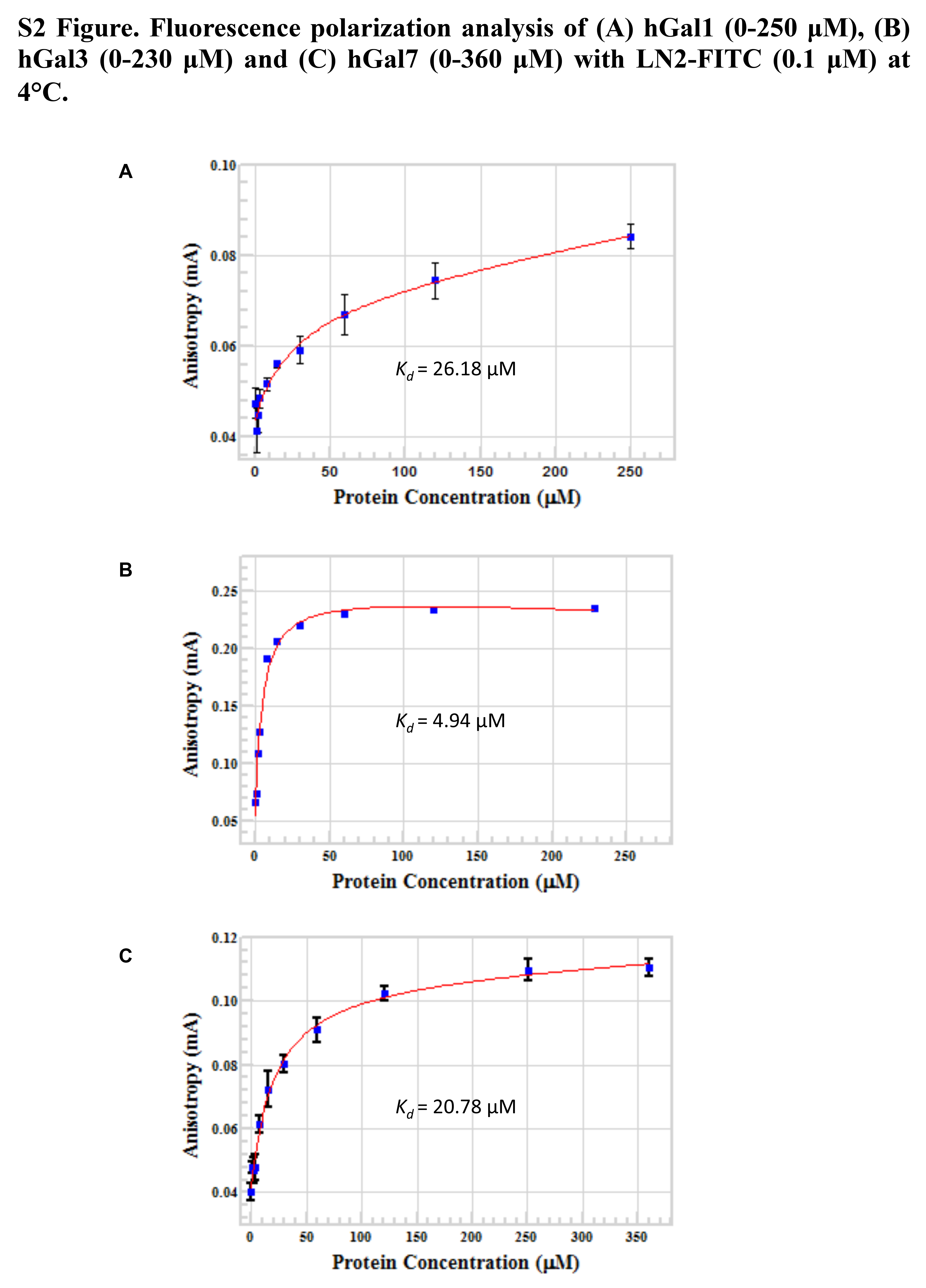

Supplement: S2 Fig — Duplicate measurements are shown for all data points. The curves represent fitting of the data by nonlinear regression to the simplified formula for a one to one interaction by Prism 5.0 software (GraphPad, San Diego, CA). Their K d values were extracted from the fitting and shown as indicated, respectively. (TIF) [file pone.0125946.s002.tif]
